# The Impact of Extended E-Learning on Emotional Well-Being of Students during the COVID-19 Pandemic in Saudi Arabia

**DOI:** 10.3390/children9010013

**Published:** 2021-12-27

**Authors:** Sehar-un-Nisa Hassan, Fahad D. Algahtani, Mohammad Raafat Atteya, Ali A. Almishaal, Ahmed A. Ahmed, Sofian T. Obeidat, Reham Mohamed Kamel, Rania Fathy Mohamed

**Affiliations:** 1 College of Public Health and Health Informatics, University of Ha’il, Ha’il 81451, Saudi Arabia; 2 Molecular Diagnostic & Personalized Therapeutic Unit, University of Ha’il, Ha’il 81451, Saudi Arabia; 3 College of Applied Medical Sciences, University of Ha’il, Ha’il 81451, Saudi Arabia; Mr.attya@uoh.edu.sa (M.R.A.); A.Almishaal@uoh.edu.sa (A.A.A.); rfathy79@gmail.com (R.F.M.); 4 Department of Social Sciences, College of Arts, University of Ha’il, Ha’il 81451, Saudi Arabia; aa.mohammed@uoh.edu.sa; 5 Department of Working with Individual and Families, Faculty of Social Work, Helwan University, Helwan 11795, Egypt; 6 Department of Basic Sciences, Preparatory Year, University of Ha’il, Ha’il 81451, Saudi Arabia; s.obeidat@uoh.edu.sa; 7 Department of Psychiatry, Cairo University, Cairo 41516, Egypt; rkamel2013@gmail.com

**Keywords:** adolescents, emotional health, e-learning, online education, stress reactions, COVID-19 pandemic, students, psychological well-being

## Abstract

Educational institutions in Saudi Arabia extended e-learning until the third semester of the academic calendar to prevent the spread of COVID-19 infection and to achieve 70% inoculation for the Saudi population. This study assesses the impact of extended e-learning and other associated stressors on the emotional health of university students in Saudi Arabia. An online cross-sectional survey collected data between the months of January–March 2021. The emotional signs of stress were measured by using a subset of items from the COVID-19 Adolescent Symptom and Psychological Experience Questionnaire (CASPE). Data about demographic variables, educational characteristics and academic performance were also collected. A regression analysis was performed to determine predictors of emotional health. A total of 434 university students including females (63%) and males (37%) provided responses. One-third of students (33%) indicated that the COVID-19 pandemic and its resulting changes including online distance studies greatly influenced their daily lives in a negative way. The regression analysis demonstrated that female students and students with average academic performance had increased vulnerability to experience emotional signs of stress (*p* < 0.05). The factors ‘Not going to university’ and ‘Not having a routine life’ were significant predictors of stress responses (*p* < 0.01) and (*p* < 0.001) respectively. E-learning during the COVID-19 pandemic made it possible for students to complete their studies as per academic calendar; simultaneously, it increased the vulnerability to experience stress, particularly for female students and students with average academic performance. These findings imply that academic advising and counseling services should be more readily available during digital studies to support at risk students.

## 1. Introduction

The severe acute respiratory syndrome coronavirus-2 (SARS-CoV-2), a new coronavirus causing acute respiratory disease subsequently termed as corona virus disease 2019 (COVID-19), was first reported in Wuhan, China in December 2019 and rapidly spread all over the world, causing a pandemic. In response to the declaration of the COVID-19 global pandemic by the World Health Organization (WHO) on 11 March 2020, the Saudi Arabian Ministry of Health (MoH) and the national public health authorities implemented very strict precautionary measures to control the transmission and spread of the disease including protocols for social distancing, a complete lockdown, hand hygiene, a travel ban and closing public and commercial aggregation. Furthermore, the Ministry of Education (MoE) in collaboration with public health authorities recommended that all nationwide educational institutions in Saudi Arabia adopt e-learning as per WHO guidelines for various countries to control the transmission and spread of the infection. In Saudi Arabia, a complete e-learning system was implemented in lieu of in-person education from March 2020 until May 2021. The COVID-19 pandemic thus disrupted the regular education system and processes, which also contributed to increased health, economic and social burdens worldwide [1].

“Online Distance Education” or ‘E-learning” refers to an institution based formal education where both students and instructors are isolated, and it utilizes interactive telecommunications systems to connect teachers, learners and other learning resources [2]. E-learning during the COVID-19 pandemic was the most sustainable way to continue studies as per the academic calendar [3]. Thus, a majority of the countries around the globe directed educational institutions to implement online studies at all levels, which prevented the spread of the COVID-19 and facilitated the continuation of the studies during the last academic year [4,5,6]. However, educational institutions, teaching staff and students faced multiple challenges in terms of execution and quick adaptation to e-learning, as demonstrated by studies conducted in the early phases of the pandemic [7,8,9]. Several cross-sectional surveys [10,11,12] and some qualitative studies [13,14] were also conducted in the early period of the pandemic to draw upon students’ and teacher’s experiences. 

During the early period of pandemic, researchers focused on assessing generalized stress responses to capture the immediate psychological responses of people and students due to the COVID-19 pandemic [5,13,15,16]. Few cross-sectional surveys of university students in Saudi Arabia measured perceived academic stress, anxiety and depression symptoms [17,18,19,20]. One study reported that students in undergraduate programs experienced high levels of anxiety, depression, insomnia and low levels of resilience during the pandemic [18], whereas some other studies reported moderate levels of depression, stress and anxiety symptoms experienced by students [12] and the general population [19].

The findings from these studies provided useful insight about the psychological repercussions of the COVID-19 pandemic on youth. Nevertheless, in the beginning of the year 2020, there were multiple factors that contributed to the generalized stress response in the masses. Some of those include fear factor at the global level, a lack of any medical intervention, high mortality statistics and people closely following the media coverage during the lockdown, which centered around the horrific effects of the pandemic. In most of the countries, including Saudi Arabia, the morbidity and mortality rates as well as the global panic situation were somewhat controlled by the end of the year 2020. The first mass vaccination program also started in December 2020 (WHO Official—COVID-19 vaccine updates) [20]. The Saudi government took strict measures to prevent the spread of COVID-19 infection and the online distance education was extended until the third term of the academic calendar. The campus education was restricted until the time when 70% of the population in Saudi Arabia received their COVID-19 vaccination. 

The current study aims at assessing the emotional experiences of university students studying through e-learning during the COVID-19 pandemic in Saudi Arabia. Our study is differentiated from prior studies in few ways. Firstly, previous studies collected data immediately after the COVID-19 outbreak in Saudi Arabia [6,15,17,21]. We collected the data for this study almost one year after of the COVID-19 outbreak in Saudi Arabia. However, social distancing measures and distance online studies were still implemented in Saudi Arabia. Secondly, the sample for this study comprised university students who continued e-learning in the third term of their academic calendar. Thirdly, this study focuses on identifying which events/changes due to the COVID-19 pandemic were considered by students as most negative. Lastly, we assessed the relative contribution of events/changes, including e-learning due to the COVID-19 pandemic, in causing emotional signs and symptoms of stress. This evidence-based knowledge will help to identify and address the contributing factors. The findings from this study will be useful to prevent the persistence of the stress from the pandemic and its implications on education and the emotional well-being of university students. 

A set of research questions considered for this analysis were:

- What are students’ perceptions about the degree of the impact of the COVID-19 pandemic on their everyday lives?
- How is the emotional well-being of male and female university students who continued e-learning in the third term of the academic calendar during the COVID-19 pandemic in Saudi Arabia?
- Which events/changes in daily life due to the COVID-19 pandemic are perceived as most negative by male and female students?
- How do these events/changes in daily life associate with the poor emotional well-being of students?

## 2. Materials and Methods

### 2.1. Study Design and Sample

A cross-sectional online survey was used to collect data, considering the adherence to the social distancing protocols and online instruction that was strictly implemented in all educational institutions during the COVID-19 pandemic in Saudi Arabia. The data were obtained from students enrolled in undergraduate programs offered by educational institutions in the Ha’il region of Saudi Arabia. The estimated sample size for this cross-sectional survey was 381 based on Fisher’s formula [22] by choosing the confidence interval at 95%, and, to account for the non-response rate, the targeted sample size was estimated to be at least 400 participants. Participation in this study was voluntary, with no compensation or incentives for participants. 

### 2.2. Survey Questionnaire

The data subset used for this research paper was collected under a wider research project aimed at assessing youth mental health and well-being during the COVID-19 pandemic in Saudi Arabia. Other than collecting data on demographic and educational characteristics, we assessed the emotional impacts of the COVID-19 pandemic and online education by employing sections from the COVID-19 Adolescent Symptom and Psychological Experience Questionnaire (CASPE) [23]. This tool was developed in the early period of the pandemic and has been used in a few studies [1,24], which demonstrates the adequate reliability and validity of the measure.

a. Demographic and educational characteristics: The survey collected data about students’ socio-demographic characteristics (gender, age, marital status, being a parent of a child and work status) and educational characteristics (field of study and academic performance). The academic performance was based on Cumulative Grade Point Average (CGPA). Students who had a CGPA of less than 2.5 were categorized as ‘low academic performance’, students who obtained a CGPA between 2.5 and 3.0 were categorized as ‘average academic performance’ and students who had a CGPA between 3.0 and 4.0 were categorized as ‘high academic performance’.
b. Negative experiences related to the COVID-19 pandemic: To assess the students’ negative experiences related to the COVID-19 pandemic, we used the first two items from the measure (CASPE) [23]. The first item assessed the degree of the impact of COVID-19 on the daily life of students. This item is phrased as “How much has the COVID-19 outbreak, and the resulting changes to daily life, affected your life in a NEGATIVE way?” The response options were on a 5-point Likert scale (Very Slightly/Not at All = 1; A little = 2; Somewhat = 3; A lot = 4; and A great deal = 5). The second item on CASPE assessed the nature of the event or change to daily life that has been associated with the most negative impact for students. Students were asked “What event or change to daily life has been the most NEGATIVE” followed by a list of events such as “having to stay at home”, “worried about someone who has or has had the coronavirus”, “not seeing friends in person”, “not going to university”, “not going to work”, not having a routine life”, “not having access to things I need”, “loss of income”. The response options were dichotomous as “Yes/No”.
c. Impact on Emotional Well-being due to changes in daily life during the COVID-19 pandemic including e-learning: To assess the emotional impacts of events/changes during the COVID-19 pandemic, including online distance studies, we used the emotional experience section from the (CASPE) [23]. Students were presented with a list of 15 items assessing emotional signs & symptoms of stress such as feelings of anxiousness, sadness, anger, worry, irritability, concern, fear, boredom, loneliness, frustration, loss of focus, having racing thoughts, being forgetful in daily activities and being disorganized. Students were asked “To what extent have the following things happened to you in the past 15 days due to events/changes in daily life, including online teaching/distance learning?” To determine the intensity of these emotional experiences, students were asked to rate each item on the on a 5-point Likert scale (Not at all = 1; Slightly = 2; Moderately = 3; Quite a bit = 4; Extremely = 5). The lowest score on the scale was 15 and the highest score was 75.

### 2.3. Data Collection

The data for this study were collected via an online survey form. The informed consent was presented to prospective participants along with a request to participate in the survey. Students who gave consent were allowed to proceed to the next sections of the questionnaire. The data collection was completed between the months of January 2020 and March 2021. This is the third semester of the academic calendar since the implementation of e-learning in educational institutions of Saudi Arabia. 

### 2.4. Data Analysis

Statistical analyses were conducted using IBM SPSS (25.0 version) for Windows (SPSS Inc, Chicago, IL, USA). The descriptive characteristics of variables under study were described using percentage values and mean scores. To determine gender difference across study variables, chi-square and *t*-test were applied by choosing the *p*-value significance *p* < 0.05. The data were checked to meet the assumptions of the regression analysis. The regression analysis was performed to determine the significant predictors of emotional signs and symptoms of stress. A *p*-value less than or equal to 0.05 and a confidence interval (CI) at 95% were considered statistically significant.

### 2.5. Ethical Approval

The research has been reviewed and approved by the Research Ethics Committee (REC) at the University of Ha’il dated: 29 December 2020 and approved by the university letter 25518//5/42). Participants were prompted to complete the questionnaire after consenting to participate in the online questionnaire.

## 3. Results

### 3.1. Demographic and Educational Characteristics

A total of 434 students completed the survey. Table 1 shows the demographic and educational characteristics of students across genders. All of the students confirmed their student status and studied full-time. Females were (63%) vs. male respondents (37%). The youngest age was 16.9 years and the oldest age was 25.8 years. For this analysis, we categorized the participants into three age groups; (21%) were below the age of 20 years; (71%) in age range of 21–23 years; and (6%) in the age range of 24–26 years. Regarding the field of study, the highest proportion of respondents were from Health and Medical Sciences (56%) and the lowest were from the discipline of Art & Humanities (13%). Approximately half of students (54%) have a CGPA > 3.0, placing them in the high academic performance category. About (21%) of the respondents are parents of a child.

### 3.2. Comparison of Male and Female Students on Emotional Well-Being Measure

To assess the impacts of events/changes in daily life during the COVID-19 pandemic, including distance education/e-learning, students were asked to rate their emotional symptoms of stress on a 5-point Likert Scale, and the mean score was calculated for each item and sum of items. The summative mean score was (M = 37.2; S.D. = 15.2). Table 2 shows that the mean score on most of the items lie in the moderate range (M = 2.28; S.D. = 1.27 to M = 2.77; S.D. = 1.38). These values show students had moderate mean scores on feelings of worry, anxious, loneliness, frustration, loss of focus, being disorganized, having racing thoughts and being forgetful in daily activities. Female students generally had statistically significant higher mean scores on all of the items assessing emotional and stress responses (*p* < 0.05) except fear of being lonely, having racing thoughts and being worried (*p* > 0.05). Overall, our findings demonstrate that the female group of students had a higher summative mean score (M = 39.2; S.D. = 16.3) vs. male students (M = 33.9; S.D. = 13.5), with the *p*-value significant at *p* < 0.001.

### 3.3. Demographic and Educational Characteristics Associated with Poor Emotional Well-Being of Students 

A regression analysis was performed to identify the characteristics of students vulnerable to experience poor emotional well-being during the COVID-19 pandemic. The results demonstrate that students with average academic performance were more likely to experience emotional symptoms of stress (β = 5.51, CI, 2.12–8.91, *p* < 0.001). Female students were more vulnerable to experience emotional symptoms of stress (β = 5.33, CI, 2.34–8.32, *p* < 0.001). Additionally, students enrolled in Health Sciences (β = 7.45, CI, −11.93–−2.96, *p* < 0.01), IT & Engineering (β = 7.19, CI, −11.73–−2.96, *p* < 0.01) and Management Sciences & Business Studies (β = 11.43, CI, −16.86–−6.01 *p* < 0.001) programs were more likely to experience stress symptoms in comparison to students studying in Arts and Humanities programs (Table 3).

### 3.4. Negative Experiences Related to the COVID-19 Pandemic

Next, we aimed to assess the student’s perceptions about the negative impact of the COVID-19 pandemic and the nature of events/changes due to the COVID-19 pandemic perceived as negative by students. In this sample, a quarter of the respondents (26%) perceived that the COVID-19 pandemic has ‘a lot’ of negative impact on their daily lives and 27 respondents (7%) considered it as ‘a great deal’, with a non-significant difference across genders (Table 4). More than two-thirds of the participants reported several sources of events/changes that have been associated with a negative impact on daily life. These included being worried about someone who has or has had COVID-19 (68%), having to stay at home (74%), not seeing friends in person (73%), not going to university (70%), not having a routine life (70%) and not having access to things needed (70%). Although these stressors were reported more by female students compared to male students, there were no significant differences across genders on most of the stressors except “not seeing friends in person” (X^2^ (1) = 16.8; *p* < 0.001) and “not going to college/university” (X^2^ (1) = 8.52; *p* < 0.01) (Table 4). These findings demonstrated that both male and female students viewed most of the events/changes to daily life due to the COVID-19 pandemic as negatively impacting their life and major sources of perceived stress. 

Between-group comparisons about the negative impact of the COVID-19 pandemic on daily life across demographics and educational characteristics are presented in Supplementary Table S1. When comparing students’ age groups, a significant negative impact of COVID-19 on daily life was observed in individuals aged between 21–25 years compared to other age groups (X^2^ (2) = 29.6, *p* < 0.001) (Supplementary Table S1). Students in Health and Medical fields reported a higher negative impact of COVID-19 on daily life than those from other colleges with statistically significant differences (X^2^ (3) = 40.7, *p* < 0.001). Furthermore, we also observed differences in students’ cumulative GPA on the impact of COVID-19 on daily life. Specifically, students with higher GPA reported a higher negative impact of COVID-19 on daily life, although the differences were not statistically different (X^2^ (3) = 18.6, *p* > 0.05) (Supplementary Table S1).

A multiple regression analysis was performed to examine the factors associated with the poor emotional well-being of students due to events/changes during the COVID-19 pandemic, including online studies (Table 5). The findings demonstrated that the changes ‘not going to university’ (β = 5.13, CI, 1.68–8.57, *p* < 0.01) and ‘not having a routine life’ (β = 8.12, CI, 4.70–11.53, *p* < 0.01) significantly increase the likelihood of emotional symptoms of stress among university students (Table 5).

## 4. Discussion

The COVID-19 pandemic may be particularly challenging for university students due to its direct impact on their daily life routines, studies, social life, future career and employment prospects. E-learning was implemented in most countries, including Saudi Arabia, to prevent the spread of the COVID-19 infection in the year 2020. However, students in Saudi Arabia had an extended e-learning period to achieve the target of 70% inoculation for the population in Saudi Arabia. The present study assessed the nature of events/changes in daily life due to the COVID-19 pandemic, including extended online studies in Saudi Arabia, which might have influenced university students’ emotional well-being. This understanding is useful to take appropriate and timely actions to prevent its implications on their academic progress and mental health. 

Our study findings demonstrated that more than one-third of students in this sample reported that COVID-19 and its resulting changes on daily life have a lot of negative influence on their everyday lives, and one-quarter of students considered it somewhat negative. These findings validate that the COVID-19 pandemic and the prolonged implementation of e-learning influenced the everyday lives of students. Educational institutions in Saudi Arabia quickly adopted online distance studies due to the advanced hardware and software infrastructure in the institutions and overall country [25]. Consequently, students had an uninterrupted academic schedule during the academic years 2020 and 2021. Regardless of its effectiveness in the continuation of the educational process, the psychological/emotional well-being of the students was influenced as validated by current study findings. The possible reason could be that, conventionally, the learning platform is tailored as a self-learning guide for students in distance studies [26]. However, as soon as the COVID-19 pandemic spread to different regions of world, educational institutions were forced to make this sudden shift to online education; thus, courses from various disciplines were not completely modified to be delivered as distance courses [27,28]. The growth of internet-based information technology and the worldwide web facilitates the quick adoption to distance education during the pandemic [4]. Nonetheless, several co-existing factors influenced the online education process, learning outcomes and students’ physical, psychological and social well-being. For instance, findings from a recent study based upon a large sample of school children in China reveals that exposure to increased screen-time during daily e-learning significantly increases the risk of myopia symptoms [29]. The study findings also recommended increased engagement in outdoor activities to mitigate this risk, which demonstrated a negative relationship with the progression of myopia symptoms in these children. Besides, the literature demonstrates that the successful outcomes of e-learning depend on personal factors, teaching processes and resources. Aspects such as personal resourcefulness, previous academic experiences, technological resources and environmental factors might determine stress responses in the university student population [9,30,31].

Our study findings show that ‘Not going to the university’ and ‘Not having a routine life’ significantly contribute to the signs and symptoms of stress experienced by students during e-learning. These findings contradict two previous studies from Saudi Arabia, which collected data during the early times of pandemic from a well-reputed higher educational institute in Saudi Arabia [3,25]. These studies reported that a larger proportion of students had positive perceptions about e-learning and were satisfied with their study experiences. It is quite probable that the timing of the cross-sectional survey influenced students’ perceptions. During the early times of pandemic, students might have demonstrated more satisfaction with online studies, which provided them with a platform to continue their studies as per the academic schedule. However, the prolonged closure of educational institutions was likely to have psychological consequences. The on-campus education provides a structured environment, social support, direct access to instructors and engagement in college sports and other competitive activities that contribute positively in terms of learning experiences, social networking and the general wellbeing of students in higher education. The reliance on distance education for longer periods deprived them of such exposure, which may generate stress responses in students, as depicted by our study findings. This is validated by a descriptive finding of our study, which showed that more than one third of students consider the COVID-19 pandemic and its resulting changes to have negatively influenced their daily lives. Findings from a previous study also imply that appropriate public health intervention is required to prevent the negative psychological impacts of increased exposure to digital devices, extended home confinement and social isolation, which are associated with e-learning exposure [32].

Our analysis also focused on examining gender differences in terms of the nature of stressors and stress responses. There was non-significant gender difference regarding the nature of stressors, which verifies that both male and female students considered ‘having to stay at home’; ‘not seeing friends in person’; ‘not going to university’; ‘not going to work’; and ‘not having a routine life’ as among the few major stressors experienced by them during the COVID-19 pandemic. However, the findings demonstrate a significant gender difference in the intensity of emotional signs and symptoms of stress. Females had statistically significant higher mean scores on all stress responses and total stress scores in comparison to male students. These findings are explicable keeping in view that the data for this study were collected from institutions located in the Ha’il region and the majority of the students enrolled in these institutions are from Ha’il city and its suburbs, which include sub-urban and rural areas. People in these regions strictly adhere to traditional social mores of Saudi society. Despite social distancing measures and distance education, male students in comparison to female students have more opportunities to go out of their homes, meet their friends and combine study options due to greater independence in terms of having their own vehicles of transportation and freedom of movement. Secondly, female students are generally more prone to experience stress, anxiety and depression symptoms, as indicated by previous studies [33,34]. Several factor may associate with the increased susceptibility of female students to poor mental health, such as economic hardships due to less employment opportunities in traditional societies, being a parent and increased exposure to adverse experiences in some regions and cultures [35]. 

Stress among university students is predictable, as demonstrated by the previous literature [36]. Eliminating studies related that stress in higher education is not feasible because university students often self-impose stress on them to exceed in academics [37]. Our study findings also showed that the fields of study and academic performance of students significantly predicted emotional signs of stress. Students enrolled in Medical Sciences, Business Studies, Information Technology and Engineering programs were more vulnerable to emotional/psychological health concerns. There is some previous evidence about the increased vulnerability of medical students for experiencing stress and depressive symptoms based upon the comparison of medical and non-medical students in Saudi Arabia [38]. The increased vulnerability of health science students to experience stress symptoms is validated by our study findings. This risk is attributable to both factors i.e., difficult course contents along with challenges in the online delivery of courses in fields of medical sciences, as supported by a few other studies during the pandemic [13,39]. 

Our study demonstrates that students with average academic performance were more likely to experience emotional signs of stress in comparison to students with low and high academic performance. The psychological literature demonstrates that a certain amount of study-related stress has been seen to some extent advantageous because it can be viewed as motivational element for a student to focus on their studies [40,41]. However, elevated levels of academic stress with low learned resourcefulness influence academic performance [42]. Positive or good stress (eustress) that a person can easily adjust to should be differentiated from the deconstructive or bad stress (distress) that negatively influences health, academic performance and general well-being [40]. Stress becomes troublesome when the academic demands outweigh the student’s capacity to cope with the situation. Furthermore, having inadequate resources to deal with the demands of the situation leads to distress [43,44]. It is quite probable that students who obtained a CGPA between 2.5–3.0 were less satisfied with their academic performance during e-learning, which might associate with emotional signs of stress. Our findings are comparable with the findings of a study that demonstrated that international students studying in universities in China during the COVID-19 pandemic experienced high levels of study stress and burnout during e-learning [45]. Furthermore, these psychological difficulties negatively associate with academic performance. The findings show that students who experience high levels of study stress and burnout had low academic performance; however, emotional intelligence was found to be a protective factor to handle the psychological pressures associated with online studies [45]. 

Another recent study tested some models to identify the risk and protective factors associated with students studying, learning and performing under stress conditions [46]. The personality factors such as being diligent, careful and having a positive outlook/approach towards self and life shield against the academic stress in students. The findings further demonstrate that burnout, procrastination and negative achievement emotions significantly associate with the academic stress experienced by university students. The extended e-learning during the COVID-19 pandemic increases this risk, where students’ likelihood of procrastinating and the long screen hours due to digital teaching may cause burnout symptoms. These impressions are comparable with our findings, which revealed that ‘not going to the college/university’ was endorsed by students as a major source of stress. The risk to experience academic stress is also attributed to the absence of direct interaction with teachers. This restricts the students’ access to a variety of support services, including direct guidance and counseling from their academic advisors. It is recommended that teachers should make appropriate adjustments during the online studies to create a sense of normalcy and control in students, which may include keeping regular contact hours and avoiding unexpected changes in the teaching process and assessment [46]. Additionally, equipping students with self-regulation through the application of internal and external regulation of learning processes can reduce the emotional consequences. Pre-COVID research also demonstrated that the constraint of personal interaction with course instructors and the dependence on self-discipline was associated with students’ satisfaction in online studies [47,48]. Overall, the current findings underline the provision of supportive counseling to students. Additionally, the provision of academic advising to students with average academic performance will be useful to prevent its repercussions on their future studies and mental well-being. Keeping in view the previous literature, which supports that emotion management and regulation positively contribute to academic performance [45,46,47,48,49], the authors of this study suggest adaptation of such trainings for students in varied educational settings, including online learning environments.

## 5. Limitations of the Study and Directions for Future Research

The aim of our study was to assess the emotional well-being of students and to identify what events/changes in daily life due to the COVID-19 pandemic in Saudi Arabia influence the emotional wellness of students who continued e-learning in the third term of their academic calendar. We used a self-report, online survey questionnaire, which was deemed to be a more appropriate approach to collect data from a large number of students during lockdown measures. However, the findings of the current study need to be interpreted while considering some limitations. Firstly, our sample is still relatively small, though it is considerably comparable with some previous studies. All of the participants were from institutions located in the Ha’il region, and student participation was voluntary and there was thus less variation in the student degrees. These factors limit the generalizability of the study findings to various disciplines and to other educational institutions with more advanced technological resources to support e-learning during the COVID-19 pandemic. Future studies could improve the study findings’ generalizability by recruiting students’ samples from more varied fields of studies, various regions of Saudi Arabia and including both public and private universities. The future research should also conduct a comparative analysis of some regional studies; for instance, a recent publication such as a large-scale dataset is being developed by researchers to assess the Jordanian university students’ psychological well-being in the context of the use of digital tools for e-learning [50]. Similar datasets can be generated for a comparative analysis, which will be used to devise effective educational and mental health policies for students in the Middle East region. 

A second limitation of study relates to the absence of exact estimates on hours of e-learning engagement, which may determine the intensity and range of the emotional reactions experienced by students. However, keeping in view the educational institution policy of a semester course load in undergraduate programs, the minimum weekly hours of engagement in e-learning is 12 h and the maximum is 19 h for scheduled courses. Thirdly, we used only one section on (CASPE) to assess the emotional signs and symptoms stress experienced by students during the COVID-19 pandemic and online distance studies. The self-report and online deployment of the survey questionnaire prevented us from doing an in-depth mental health assessment. Future research can improve the study design by collecting qualitative data and the use of wide-ranging psychological tools to validate the psychological repercussions among university students due to the extended reliance on e-learning during the COVID-19 pandemic. Future research should focus on collecting longitudinal data to validate the impact of e-learning on academic performance and the identification of specific factors at an individual level, institutional level and environmental level, which positively or negatively influence the learning process and learning outcomes for university students. Additionally, we did not collect the information about general health and if participants were infected or not infected or with infected family members, which have been shown to be positively associated with higher levels of stress. Despite some of these limitations, our findings provide useful insight into the impact of the subsequent wave of the COVID-19 pandemic on the psychological well-being and its sources among university students.

## 6. Conclusions

E-learning during the COVID-19 pandemic made it possible for university students to complete their studies as per the academic calendar; nonetheless, factors such as ‘not going to the university’ and ‘not having a routine life’ increased their vulnerability to experience poor emotional well-being. The findings imply that academic advising and counseling services should be more readily available during digital studies to support students. Additionally, academic advisors and university counseling services should expand supportive counseling services for female students and students with average academic performance to improve their online learning experiences and mental well-being. 

## Figures and Tables

**Table 1:** Demographics and educational characteristics of students across genders (*n* = 434).

		Female *n*(%)	Male *n* (%)	Chi-Square Value and Significance
Variables	Total Count	272 (63%)	162 (37%)	
Age Categories	<20 years (*n* = 93; 22%)	77 (29%)	16 (10%)	X^2^ (2) = 28.6 ***
	21–23 years (*n* = 313; 71%)	186 (68%)	127 (78%)	
	24–26 years (*n* = 28; 6%)	9 (3%)	19 (12%)	
Field of Study	Health /Medical Sciences (*n* = 243; 56%)	180(66%)	63 (39%)	X^2^ (3) = 101.6 ***
	IT & Engineering (n = 67; 15%)	6 (2%)	61 (38%)	
	Business & Management Sciences (*n* = 69; 16%)	43 (16%)	26 (16%)	
	Art & Humanities (*n* = 55; 13%)	43 (16%)	12 (7%)	
CGPA	Low Academic Performance (*n* = 80; 19%)	53 (21%)	27 (17%)	X^2^ (2) = 3.78, (ns)
	Average Academic Performance (*n* = 120; 28%)	82 (30%)	38 (24%)	
	High Academic Performance (*n* = 234; 54%)	137 (59%)	97 (60%)	
Employed	Yes (*n* = 213; 49%)	152 (56%)	60 (37%)	X^2^ (1) = 14.4 ***
	No (*n* = 221; 51%)	120(44%)	102 (63%)	
Parent of a child	Yes (*n* = 90; 21%)	57 (21%)	33 (20%)	X^2^ (1) = 0.02, (ns)
	No (*n* = 344; 79%)	215(79%)	129 (80%)	

Level of significance *** *p* < 0.001; ns = non-significant.

**Table 2:** Emotional signs and symptoms of stress in the total sample and across genders (n = 434).

Emotional Signs of Stress	Total Sample	Female	Male	t-Statistic & *p*-Value
	Mean (S.D.)	Mean (S.D.)	Mean (S.D.)	
Anxious	2.50 (1.38)	1.68 (1.43)	1.20 (1.23)	t (432) = 3.68 ***
Angry	2.28 (1.27)	1.49 (1.35)	0.93 (1.05)	t (432) = 4.81 ***
Afraid	2.56 (1.33)	1.69 (1.37)	1.34 (1.23)	t (432) = 2.64 **
Sad	2.36 (1.33)	1.49 (1.39)	1.15 (1.20)	t (432) = 42.59 *
Worried	2.75 (1.40)	1.82 (1.45)	1.63 (1.29)	t (432) = 1.36 (ns)
Irritable	2.31 (1.30)	1.49 (1.39)	1.01 (1.08)	t (432) = 3.98 ***
Concerned	2.39 (1.21)	1.53 (1.26)	1.15 (1.07)	t (432) = 3.32 **
Bored	2.77 (1.38)	1.89 (1.41)	1.58 (1.34)	t (432) = 2.23 *
Lonely	2.45 (1.45)	1.53 (1.41)	1.31 (1.33)	t (432) = 1.53 (ns)
Frustrated	2.42 (1.31)	1.53 (1.41)	1.24 (1.12)	t (432) = 2.31 *
Loss of focus	2.60 (1.36)	1.81 (1.41)	1.27 (1.25)	t (432) = 3.96 ***
Having racing thoughts	2.38 (1.29)	1.44 (1.27)	1.27 (1.32)	t (432) = 1.34 (ns)
Forgetful in daily activities	2.46 (1.26)	1.59 (1.32)	1.23 (1.13)	t (432) = 2.91 **
Unable to plan activities	2.52 (1.30)	1.65 (1.37)	1.28 (1.15)	t (432) = 3.01 **
Disorganized	2.50 (1.27)	1.61 (1.32)	1.30 (1.17)	t (432) = 2.47 *
Total Score on Scale	37.2 (15.4)	39.2 (16.3)	33.9 (13.5)	t (432) = 3.67 ***

Level of significance *** *p* < 0.001; ** *p* < 0.01; * *p* < 0.05; ns = non-significant.

**Table 3:** Demographic and educational characteristics associated with the poor emotional well-being of students on extended e-learning in Saudi Arabia during the COVID-19 pandemic (N = 434).

		Unstandardized Coefficients		Standardized Coefficients		95% Confidence Interval for β	
Variables	Categories	β	Std. Error	Beta	t	Lower Bound	Upper Bound
Gender	Constant	33.91	1.21		28.12	31.51	36.22
	Female	5.33	1.52	0.16	3.51 ***	2.34	8.32
	Male (Reference Category)						
Age Categories	Constant	31.64	2.94		9.35	29.85	32.42
	<20 years	1.01	3.35	0.27	0.763 (ns)	−5.58	7.61
	21–23 years	0.53	3.07	0.01	0.17 (ns)	−5.51	6.57
	24–26 years (Reference Category)						
Fields of Study	Constant	39.34	2.06		24.23	29.29	40.97
	Health Sciences	7.45	2.28	−0.23	3.26 **	−11.93	−2.96
	IT & Engineering	7.19	2.78	−0.16	2.58 **	−12.66	−1.72
	Management Sciences & Business Studies	11.43	2.76	−0.26	4.13 ***	−16.86	−6.01
	Arts & Humanities (Reference Category)						
Academic Performance	(Constant)	32.69	1.01		20.57	28.71	34.66
	Low Academic Performance	0.14	1.99	0.01	0.07 (ns)	−3.77	4.06
	Average Academic Performance	5.51	1.72	0.15	3.19 **	2.12	8.91
	High Academic Performance (Reference Category)						
Employment	Constant	38.01	1.04		36.01	35.95	40.06
	Yes	−1.55	1.49	−0.05	−1.04 (ns)	−4.49	1.37
	No (Reference Category)						
Married	Constant	36.3	0.85		42.1	30.67	34.02
	Yes	−0.44	1.76	−0.12	−0.25 (ns)	−3.91	3.01
	No (Reference Category)						
Parent of child	Constant	37.1	0.83		44.26	35.46	38.76
	Yes	0.61	1.84	0.01	0.33 (ns)	−3.01	4.23
	No (Reference Category)						

Level of significance *** *p* < 0.001; ** *p* < 0.01; ns = non-significant.

**Table 4:** Negative Impact of the COVID-19 pandemic on the daily lives of university students (n = 434).

Negative Impact of the COVID-19 Pandemic on Daily Life (M = 3.01; S.D. = 1.14)	Total n (%)	Female n (%)	Male n (%)	Chi-Square Value and Significance
Very Slight or Not at All	57 (13%)	35 (13%)	22 (14%)	X^2^ (4) = 4.5, (ns)
A little	127 (29%)	80 (29%)	47 (29%)	
Somewhat	108 (25%)	65 (24%)	43 (26%)	
A lot	115 (26%)	70 (26%)	45 (28%)	
A great deal	27 (7%)	22 (8%)	5 (3%)	
**Event/change to daily life due to the COVID-19**				
Worried about someone who has or has had the coronavirus	295 (68%)	188 (70%)	107 (66%)	X^2^ (1) = 0.43, (ns)
Having to stay at home	321 (74%)	198 (73%)	123 (76%)	X^2^ (1) = 0.51, (ns)
Not seeing friends in person	317 (73%)	217 (79%)	100 (62%)	X^2^ (1) = 16.8 ***
Not going to university	304 (70%)	204 (75%)	100 (61%)	X2 (1) = 8.52 **
Not going to work	225 (52%)	155 (57%)	70(43%)	X^2^ (1) = 7.17, (ns)
Not having a routine life	305 (70%)	188 (69%)	117 (72%)	X^2^ (1) = 0.468; (ns)
Not having access to things, I need	303 (69%)	184 (68%)	119 (72%)	X^2^ (1) = 1.62; (ns)
Loss of income	239 (55%)	158 (58%)	81 (50%)	X^2^ (1) = 2.68; (ns)

Level of significance *** *p* < 0.001; ** *p* < 0.01; ns = non-significant.

**Table 5:** Multiple regression analysis to examine the factors associated with the poor emotional well-being of students due to events/changes during the COVID-19 pandemic, including online studies (*n* = 434).

	Unstandardized Coefficients		Standardized Coefficients	t	95% Confidence Interval for β	
	β	Std. Error	Beta		Lower Bound	Upper Bound
Constant	23.75	2.17		10.91	19.48	28.03
Worried about someone for coronavirus	1.25	1.59	0.03	0.78 (ns)	−1.87	4.39
Having to stay at home	1.35	1.71	0.03	0.79 (ns)	−2.01	4.72
Not seeing friends in person	2.18	1.67	0.06	1.31 (ns)	−1.11	5.47
Not going to university	5.13	1.75	0.15	2.92 **	1.68	8.57
Not going to work	−1.27	1.65	−0.04	−0.77 (ns)	−4.53	1.98
Not having a routine life	8.12	1.73	0.23	4.67 ***	4.70	11.53
Not having access to things, I need	3.12	1.78	0.09	1.75 (ns)	−0.38	6.64
Loss of income	−1.40	1.59	−0.04	−0.88 (ns)	−4.52	1.72

Level of significance *** *p* < 0.001; ** *p* < 0.01; ns = non-significant.

## Data Availability

The sub-set of the data presented in this study are available on request from the corresponding authors. The data are not publicly available due to data privacy issues which was collected under youth health and well-being project.

## Supplementary Materials

The following supporting information can be downloaded at: https://www.mdpi.com/article/10.3390/children9010013/s1, Table S1: Crosstab analysis between demographic variables and the negative impact of the COVID-19 pandemic on daily life (*n* = 434).

## References

[1] Cohen Z.P., Cosgrove K.T., DeVille D.C., Akeman E., Singh M.K., White E., Stewart J.L., Aupperle R.L., Paulus M.P., Kirlic N. (2021). The Impact of COVID-19 on Adolescent Mental Health: Preliminary Findings from a Longitudinal Sample of Healthy and at-Risk Adolescents. Front. Pediatr..

[2] Simonson M., Schlosser L.A. (2009). Distance Education 3rd Edition: Definition and Glossary of Terms.

[3] Meccawy M., Meccawy Z., Alsobhi A. (2021). Teaching and Learning in Survival Mode: Students and Faculty Perceptions of Distance Education during the COVID-19 Lockdown. Sustainability.

[4] Huang R., Tlili A., Chang T.-W., Zhang X., Nascimbeni F., Burgos D. (2020). Disrupted Classes, Undisrupted Learning during COVID-19 Outbreak in China: Application of Open Educational Practices and Resources. Smart Learn. Environ..

[5] Armstrong-Mensah E., Ramsey-White K., Yankey B., Self-Brown S. (2020). COVID-19 and Distance Learning: Effects on Georgia State University School of Public Health Students. Front. Public Health.

[6] Tanveer M., Bhaumik A., Hassan S., Ul Haq I. (2020). COVID-19 Pandemic, Outbreak Educational Sector and Students Online Learning in Saudi Arabia. J. Entrep. Educ..

[7] Coman C., Țîru L.G., Meseșan-Schmitz L., Stanciu C., Bularca M.C. (2020). Online Teaching and Learning in Higher Education during the Coronavirus Pandemic: Students’ Perspective. Sustainability.

[8] Müller A.M., Goh C., Lim L.Z., Gao X. (2021). COVID-19 Emergency ELearning and Beyond: Experiences and Perspectives of University Educators. Educ. Sci..

[9] Abou-Khalil V., Helou S., Khalifé E., Chen M.A., Majumdar R., Ogata H. (2021). Emergency Online Learning in Low-Resource Settings: Effective Student Engagement Strategies. Educ. Sci..

[10] Gonçalves S.P., Sousa M.J., Pereira F.S. (2020). Distance Learning Perceptions from Higher Education Students—The Case of Portugal. Educ. Sci..

[11] Ferraro F.V., Ambra F.I., Aruta L., Iavarone M.L. (2020). Distance Learning in the COVID-19 Era: Perceptions in Southern Italy. Educ. Sci..

[12] Khoshaim H.B., Al-Sukayt A., Chinna K., Nurunnabi M., Sundarasen S., Kamaludin K., Baloch G.M., Hossain S.F.A. (2020). How Students in the Kingdom of Saudi Arabia Are Coping with COVID-19 Pandemic. J. Public Health Res..

[13] Lovrić R., Farčić N., Mikšić Š., Včev A. (2020). Studying During the COVID-19 Pandemic: A Qualitative Inductive Content Analysis of Nursing Students’ Perceptions and Experiences. Educ. Sci..

[14] Aldon G., Cusi A., Schacht F., Swidan O. (2021). Teaching Mathematics in a Context of Lockdown: A Study Focused on Teachers’ Praxeologies. Educ. Sci..

[15] AlAteeq D.A., Aljhani S., AlEesa D. (2020). Perceived Stress among Students in Virtual Classrooms during the COVID-19 Outbreak in KSA. J. Taibah Univ. Med. Sci..

[16] Wang X., Hegde S., Son C., Keller B., Smith A., Sasangohar F. (2020). Investigating Mental Health of US College Students during the COVID-19 Pandemic: Cross-Sectional Survey Study. J. Med. Internet Res..

[17] Khoshaim H.B., Al-Sukayt A., Chinna K., Nurunnabi M., Sundarasen S., Kamaludin K., Baloch G.M., Hossain S.F.A. (2020). Anxiety Level of University Students during COVID-19 in Saudi Arabia. Front. Psychiatry.

[18] Alyoubi A., Halstead E.J., Zambelli Z., Dimitriou D. (2021). The Impact of the COVID-19 Pandemic on Students’ Mental Health and Sleep in Saudi Arabia. Int. J. Environ. Res. Public Health.

[19] Algahtani F.D., Hassan S.-N., Alsaif B., Zrieq R. (2021). Assessment of the Quality of Life during COVID-19 Pandemic: A Cross-Sectional Survey from the Kingdom of Saudi Arabia. Int. J. Environ. Res. Public Health.

[20] World Health Organization (WHO) Official-COVID-19 Vaccine Updates. https://www.who.int/emergencies/diseases/novel-coronavirus-2019/covid-19-vaccines.

[21] Hoq M.Z. (2020). E-Learning during the Period of Pandemic (COVID-19) in the Kingdom of Saudi Arabia: An Empirical Study. Am. J. Educ. Res..

[22] Dattalo P. (2008). Determining Sample Size: Balancing Power, Precision, and Practicality.

[23] Ladouceur C.D. (2020). COVID-19 Adolescent Symptom & Psychological Experience Questionnaire.

[24] Porter B.M., Douglas I.J., Larguinho T.L., Aristizabal M., Mitchell M.E., Roe M.A., Church J.A. (2021). Examination of Pre-Pandemic Measures on Youth Well-Being During Early Stages of the COVID-19 Pandemic. Biol. psychiatry Glob. Open Sci..

[25] Alsmadi M.K., Al-Marashdeh I., Alzaqebah M., Jaradat G., Alghamdi F.A., Mohammad R.M.A., Alshabanah M., Alrajhi D., Alkhaldi H., Aldhafferi N. (2021). Digitalization of Learning in Saudi Arabia during the COVID-19 Outbreak: A Survey. Inform. Med. Unlocked.

[26] Xiaochun X. (2002). Guide Research on Autonomous Learning in Distance Education. China Distance Educ.

[27] Al Lily A.E., Ismail A.F., Abunasser F.M., Alqahtani R.H.A. (2020). Distance Education as a Response to Pandemics: Coronavirus and Arab Culture. Technol. Soc..

[28] García-Alberti M., Suárez F., Chiyón I., Mosquera Feijoo J.C. (2021). Challenges and Experiences of Online Evaluation in Courses of Civil Engineering during the Lockdown Learning Due to the COVID-19 Pandemic. Educ. Sci..

[29] Liu J., Chen Q., Dang J. (2021). Examining Risk Factors Related to Digital Learning and Social Isolation: Youth Visual Acuity in COVID-19 Pandemic. J. Glob. Health.

[30] Li X., Fu P., Fan C., Zhu M., Li M. (2021). COVID-19 Stress and Mental Health of Students in Locked-Down Colleges. Int. J. Environ. Res. Public Health.

[31] Jan S.K. (2015). The Relationships between Academic Self-Efficacy, Computer Self-Efficacy, Prior Experience, and Satisfaction with Online Learning. Am. J. Distance Educ..

[32] Liu J., Li B., Chen Q., Dang J. (2021). Student Health Implications of School Closures during the COVID-19 Pandemic: New Evidence on the Association of e-Learning, Outdoor Exercise, and Myopia. Healthcare.

[33] Gelles L.A., Lord S.M., Hoople G.D., Chen D.A., Mejia J.A. (2020). Compassionate Flexibility and Self-Discipline: Student Adaptation to Emergency Remote Teaching in an Integrated Engineering Energy Course during COVID-19. Educ. Sci..

[34] Bayram N., Bilgel N. (2008). The Prevalence and Socio-Demographic Correlations of Depression, Anxiety and Stress among a Group of University Students. Soc. Psychiatry Psychiatr. Epidemiol..

[35] Misigo B.L. (2015). Gender Difference in the Perceived Level of Stress and Coping Strategies among University Students in Kenya: A Case of Public Universities. Int. Acad. J. Soc. Sci. Educ..

[36] Calvarese M. (2015). The Effect of Gender on Stress Factors: An Exploratory Study among University Students. Soc. Sci..

[37] Douki S., Zineb S.B., Nacef F., Halbreich U. (2007). Women’s Mental Health in the Muslim World: Cultural, Religious, and Social Issues. J. Affect. Disord..

[38] Al-Dabal B.K., Koura M.R., Rasheed P., Al-Sowielem L., Makki S.M. (2010). A Comparative Study of Perceived Stress among Female Medical and Non-Medical University Students in Dammam, Saudi Arabia. Sultan Qaboos Univ. Med. J..

[39] O’Byrne L., Gavin B., Adamis D., Lim Y.X., McNicholas F. (2021). Levels of Stress in Medical Students Due to COVID-19. J. Med. Ethics..

[40] Hamaideh S.H. (2011). Stressors and Reactions to Stressors among University Students. Int. J. Soc. Psychiatry.

[41] McEwen B.S. (1998). Protective and Damaging Effects of Stress Mediators. N. Engl. J. Med..

[42] Struthers C.W., Perry R.P., Menec V.H. (2000). An Examination of the Relationship among Academic Stress, Coping, Motivation, and Performance in College. Res. High. Educ..

[43] Akgun S., Ciarrochi J. (2003). Learned Resourcefulness Moderates the Relationship between Academic Stress and Academic Performance. Educ. Psychol..

[44] McEwen B.S. (2005). Stressed or Stressed out: What Is the Difference?. J. Psychiatry Neurosci..

[45] Alam F., Yang Q., Bhutto M.Y., Akhtar N. (2021). The Influence of E-Learning and Emotional Intelligence on Psychological Intentions: Study of Stranded Pakistani Students. Front. Psychol..

[46] de la Fuente J. (2021). A Path Analysis Model of Protection and Risk Factors for University Academic Stress: Analysis and Psychoeducational Implications for the COVID-19 Emergency. Front. Psychol..

[47] Pozos-Radillo B.E., de Lourdes Preciado-Serrano M., Acosta-Fernández M., de los Ángeles Aguilera-Velasco M., Delgado-García D.D. (2014). Academic Stress as a Predictor of Chronic Stress in University Students. Psicol. Educ..

[48] Zakariah Z., Hashim R.A., Musa N. (2016). Motivation, Experience and Satisfaction among Adult Learners with Fully Online Web-Based Courses. http://hdl.handle.net/11599/2548.

[49] Wang C.-H., Shannon D.M., Ross M.E. (2013). Students’ Characteristics, Self-Regulated Learning, Technology Self-Efficacy, and Course Outcomes in Online Learning. Distance Educ..

[50] Haider A.S., Al-Salman S. (2020). Dataset of Jordanian University Students’ Psychological Health Impacted by Using e-Learning Tools during COVID-19. Data Br..

