# Supplementary material for: The Impact of Extended E-Learning on Emotional Well-Being of Students during the COVID-19 Pandemic in Saudi Arabia
Source: Children (Basel). 2021 Dec 27;9(1):13. doi: 10.3390/children9010013 (PMC8774542; doi:10.3390/children9010013)
Supplement: Supplementary file 1 [file children-09-00013-s001.zip › children-1496152-supplementary.pdf]

Supplementary Table

Table S1. Crosstab analysis between demographic variables and negative impact of the COVID-19 pandemic on daily life (N = 434).

**Negative impact of the COVID-19 pandemic on daily life**		**Not at All n (%)**	**A Little n (%)**	**Somewhat n (%)**	**A lot n (%)**	**A great deal n (%)**	**Chi-square value and significance**
Age Categories	<20 years	3 (3.2%)	33 (35%)	21 (22%)	27 (29%)	9 (10%)	$X^2(2)=29.6^{***}$
	21-23 years	51(16%)	88 (28%)	75 (24%)	86 (27%)	13 (4%)	
	24-26 years	3 (11%)	6 (21%)	12 (43%)	2 (7%)	5 (18%)	
Field of Study	Health Sciences	25(10%)	62 (25%)	74 (30%)	62 (25%)	20 (8%)	$X^2(3)=40.7^{***}$
	IT & Engineering	3 (4%)	27 (40%)	7 (10%)	26 (38%)	4 (6%)	
	Business & Management Sciences	18(26%)	22 (32%)	13 (19%)	13 (19%)	3 (4%)	
	Arts & Humanities	11(20%)	16 (30%)	14 (25%)	14 (25%)	0 (0%)	
Academic performance	Low Academic Performance	17(21%)	23 (29%)	17 (21%)	18 (23%)	5 (6%)	$X^2(2)=10.2$ (ns)
	Average Academic Performance	14(12%)	43 (36%)	27 (23%)	30 (25%)	6 (5%)	
	High Academic Performance	26(11%)	61 (26%)	64 (27%)	67 (29%)	16 (7%)	
Employment	Yes	31(14%)	71 (33%)	40 (19%)	49 (23%)	22 (10%)	$X^2(1)=22.5^{***}$
	No	26(12%)	56 (25%)	68 (31%)	66 (30%)	5 (2%)	
Married	Yes	31(30%)	21 (21%)	26 (25%)	11 (11%)	13 (13%)	$X^2(1)=55.2^{***}$
	No	26 (8%)	106(31%)	82 (25%)	104(31%)	14 (4%)	
Parent of a child	Yes	24(26%)	17 (19%)	26 (29%)	10 (11%)	13 (14%)	$X^2(1)=43.2^{***}$
	No	33(10%)	110(32%)	82 (23%)	105(31%)	14 (4%)	

Levels of significance=****p*<0.001; ***p*<0.01, **p*<0.05; ns=non-significant
